# Health Literacy in Web-Based Health Information Environments: Systematic Review of Concepts, Definitions, and Operationalization for Measurement

**DOI:** 10.2196/10273

**Published:** 2018-12-19

**Authors:** Anna-Maija Huhta, Noora Hirvonen, Maija-Leena Huotari

**Affiliations:** 1 Department of Information Studies Faculty of Humanities University of Oulu Oulu Finland; 2 Medical Research Center Oulu Oulu University Hospital and University of Oulu Oulu Finland; 3 Department of Information Studies Faculty of Social Sciences, Business and Economics Åbo Akademi University Turku Finland

**Keywords:** health literacy, consumer health information, internet, review, systematic

## Abstract

**Background:**

Health literacy research seems to lack a consensus on what aspects to include into *literacy* in the context of health and on how to operationalize these concepts for measurement purposes. In addition to health literacy, several other concepts, such as electronic health (eHealth) literacy and mental health literacy, have been developed across disciplines. This study examines how these different concepts are used when studying health-related competencies in Web contexts.

**Objective:**

This study systematically reviews health literacy concepts and definitions and their operationalization in studies focused on Web-based health information environments.

**Methods:**

A systematic literature search was conducted in April 2016 in 6 electronic databases with a limitation to articles in English published between January 2011 and April 2016. Altogether, 1289 unique records were identified and screened according to the predefined inclusion criteria: (1) original, peer-reviewed research articles written in English; (2) the topic of the article concerned literacy in the context of health; (3) informants of the study were lay people, not health professionals or students of the field; and (4) the focus of the study was placed on an Web-based information environment. In total, 180 full texts were screened, of which 68 were included in the review. The studies were analyzed with an emphasis on the used health literacy concepts and measures.

**Results:**

On the basis of the included studies, several concepts are in use when studying health-related literacy in Web environments, eHealth literacy and health literacy being the most common ones. The reviewed studies represent a variety of disciplines, but mostly medical sciences. Typically, quantitative research methods are used. On the basis of the definitions for health literacy, 3 thematic categories were identified: general and skill-based, multidimensional, and domain-specific health literacy. Most studies adopted a domain-specific concept, followed by the ones that used a general and skill-based concept. Multidimensional concepts occurred least frequently. The general health literacy concepts were usually operationalized with reading comprehension measures, the domain-specific concepts with self-efficacy measures, and multidimensional concepts with several types of measures. However, inconsistencies in operationalization were identified.

**Conclusions:**

The results show that in studies conducted in Web-based information environments, several different health literacy concepts are in use, and there is no clear consensus on the definitions for these concepts. Future studies should place emphasis on the conceptual development of health literacy in Web contexts to gain better results on operationalization for measurement. Researchers are encouraged to provide clear operational definitions for the concepts they use to ensure transparency in reporting.

## Introduction

### Background

The contemporary digital information environment challenges our understanding of what it means to be literate. The fast and free flow of information on the internet offers multiple ways to communicate, but it can also challenge with overload of information and loss of authority and identity [1]. Exercising critical thinking and employing information and digital literacies are ways to reduce the effects of information overload [2]. These types of literacies usually refer to a diverse set of competencies, skills, and strategies vital for acting in multimodal and transforming information environments. In the context of Web-based health information, these competencies are essential as the amount of health information is rapidly increasing and the possibility to encounter misinformation is apparent.

The concept of health literacy has been widely used to address literacy competencies required in health settings. A recent definition [3] describes health literacy as a concept that recognizes people’s different capacities to find, understand, and use health information as well as the different life experiences that shape peoples’ willingness and confidence to do these tasks. According to the World Health Organization (WHO) [4], health literacy regards the environmental, political, and social factors that determine health, and it is gained through comprehensive health education at the individual and community levels. Both the concept of health literacy and the means to measure it have been under development for over three decades. Yet, the research on the phenomena seems to lack a consensus on what aspects to include into *literacy* in the context of health and on how to operationalize it for measurement purposes [5-8].

On the basis of earlier reviews, health literacy is typically understood as individuals’ functional skills, such as reading comprehension and numeracy [9] that are assessed in clinical settings [5], and the research is conducted predominantly within medical sciences [10]. More recently, however, research on health literacy–associated issues has been conducted in several other disciplines and subconcepts and related concepts have emerged [11,12]. Although the definitions have unique elements, especially the most recent definitions for health literacy overlap substantially [3]. The digital context that has changed the ways people communicate has been taken into account in the definitions of the concept only recently and, thus, needs to be investigated further.

The aim of this study was to increase understanding of the health literacy concepts that are used as well as their definitions and operationalization in Web-based information environments. The purpose was to provide a synthesis of their use in a sample of studies published between the years 2011 and 2016.

### From Health Literacy to Electronic Health Literacy

Contemporary discussion on health literacy reveals that there is no consensus on the definition of the concept [5,10,13,14]. For instance, the attributes included in the concept [10] and the distinction between basic functional health literacy, communicative or interactive health literacy, and critical health literacy have been debated [14]. Mårtensson and Hensing [9] note that the research on health literacy is heterogeneous and identify 2 perspectives: health literacy as a polarized phenomenon focused on the extremes of high and low and health literacy as a multidimensional concept that acknowledges the broadness of skills in interaction with social and cultural contexts. These definitions emphasize the interactive and critical skills needed to use information for making appropriate health decisions [9]. They also consider multiple settings and recognize that there are both social and individual components to the concept [3].

The internet and the new digital tools for seeking, communicating, and using information have become embedded in the social actions of people since the 1990s. Moreover, the growing interest in consumer health and digital solutions to tailor health information for electronic health (eHealth) purposes has increased research and generated new conceptualizations for health literacy. The concept of eHealth literacy by Norman and Skinner [15] was one of the first attempts to capture the meaning of health literacy in the digital context. The definition draws on Eng’s [16] definition of eHealth as “the use of emerging information and communication technology, especially the internet, to improve or enable health and health care.” However, Norman and Skinner [15] add to it by stating that “[c]onsumer eHealth requires basic reading and writing skills, working knowledge of computers, a basic understanding of science, and an appreciation of the social context that mediates how online health information is produced, transmitted, and received.”

The definition of eHealth literacy by Norman and Skinner [15] has been criticized for not fully describing the competencies essential in digital environments [17-19]. Gilstad [18] notes that the concept lacks the notions of contextual and cultural literacy and communicative expertise as central literacy competencies. There are several new definitions proposed for the concept. For example, Griebel et al [19] recently proposed a definition of eHealth literacy that encompasses aspects of interactivity, the dynamic evolvement of literacy, changing information practices of individuals, and the integration of technology aspects. The authors note that there are several models describing eHealth literacy but also that there is a lot of research that deals with the themes related to eHealth literacy but uses other terms [19]. Typically, health literacy is seen as an umbrella concept that covers other concepts such as eHealth literacy and mental literacy. However, the hierarchy is not entirely clear. For example, health information literacy, a concept used in information science, can be seen as a related rather than a subconcept to health literacy as it combines the concepts of health literacy and information literacy [20]. In this study, we do not focus on the hierarchical relationships of these concepts and use the phrase *health literacy concepts* to refer to all health-related literacy concepts.

### Measuring Health Literacy

The first health literacy assessment tools were designed to measure the functional health literacy of individuals in clinical settings [21]. The basis of these measures is on the definitions of health literacy that present individuals’ reading comprehension and numeracy as central competencies when dealing with medical texts. Therefore, these measures have been criticized for capturing only a narrow spectrum of the conception of health literacy [5,11,22]. Another way to assess health literacy is to measure the level of health knowledge of individuals. Usually, these measures are content- and context-based knowledge tests that have been developed in and for the use of clinical settings [11]. The more recent measures for health literacy consider individuals’ self-reported abilities or self-efficacy as an indicator of health literacy. These measures usually aim to detect the self-perceived abilities of the individual to, for example, collect, communicate, and evaluate health information [23] or to rate the individuals’ ability to understand health-related material [24]. However, the risk of assessing merely self-efficacy or behavior instead of health literacy is considered to be a major disadvantage of self-reported health literacy measures [11].

Altin et al [25] reviewed generic health literacy instruments and categorized them by their measurement modes (print, oral, numeracy, and multimodal) and their measurement approaches (objective, subjective, mixed, and multidimensional construct). The review indicated that more than two-thirds of the generic health literacy instruments were based on multidimensional constructs of health literacy. Moreover, it was shown that there is a trend toward mixing objective and subjective measurement approaches. In addition, a third of the reviewed instruments were based on existing functional literacy screeners. O’Neill et al [26] reviewed self-administered health literacy instruments and discovered that the majority of the instruments measured general health literacy, whereas one-third of them measured condition- or context-specific health literacy (see also [22]). Therefore, it was suggested that for the instruments to progress, more research should be focused on the investigation and elaboration of the construct of health literacy itself [26,27]

A systematic review on eHealth literacy measures [28] found that all the identified measures were based on self-report and measured the self-efficacy of individuals. The authors identified 3 concept-based eHealth literacy measurement tools and 5 dual-design tools that comprised individual measures of health literacy and digital literacy. The dual-design measurement tools did not intend to measure eHealth literacy but ended up doing so by including the main components of the concept [28]. An overview of the recent eHealth literacy research [29] indicates that although international research has been conducted, the tools to measure eHealth literacy lack acknowledgment of different personal backgrounds influencing the measured competencies, such as social and cultural factors. Griebel et al [19] criticize the eHealth literacy community for missing an agreement on how to measure eHealth literacy. Accordingly, it is stated that the new tools should consider the earlier research and create a well-founded theoretical basis to place eHealth literacy into broader context [19].

### Objectives

Earlier reviews have focused on: (1) the definitions and measures of the concepts of health literacy [5,6,9,11,12,25,26], eHealth literacy [28,29], and critical health literacy [13,14] and their (2) operationalization in a specific demographic group, for example, adolescents [30-32] and older adults [33], or in a specific context, for example, eHealth service use [34].

This systematic review contributes to these earlier reviews by synthesizing health literacy research conducted in *Web-based information environments* and in *different disciplines*. It differs from the earlier reviews as it reviews not only the definitions of different health literacy concepts but also the measures used to operationalize these concepts in empirical studies. By elaborating remarks made in previous literature about the conceptions of health literacy, the following objectives were set:

1. To categorize thematically the definitions of health literacy and related concepts used in empirical studies focused on Web-based information environments.
2. To examine the operationalization of the concepts within these thematic categories.

## Methods

### Data Sources and Search Strategy

This systematic review follows the Preferred Reporting Items for Systematic Reviews and Meta-Analyses (PRISMA) [35]. The review is interpretive [36] and emphasizes the integration of studies across different disciplines to create a synthesis of the data. A search strategy was developed to identify articles examining health literacy or related concepts in a Web-based information environment. Overall, 6 academic databases were searched on April 14, 2016. The databases were Library and Information Science Abstracts, Applied Social Sciences Index and Abstracts, Education Resources Information Center, US National Library of Medicine premier bibliographic database (MEDLINE), Library and Information Science and Technology Abstracts, and the Cumulative Index to Nursing and Allied Health Literature. The search terms used covered 3 domains, “web,” “health,” and “literacy,” including related terms. The search was limited to title and abstract and to peer-reviewed articles published in English between years 2011 and 2016. This time span was chosen to provide a sample of studies published during a period within which Web information seeking [37] and the use of social media [38] have increased considerably. This tight time frame enabled reviewing a manageable sample of studies. A broader time frame would have required a narrower search strategy. The search strategy is reported in detail in Multimedia Appendix 1.

**Figure 1:**
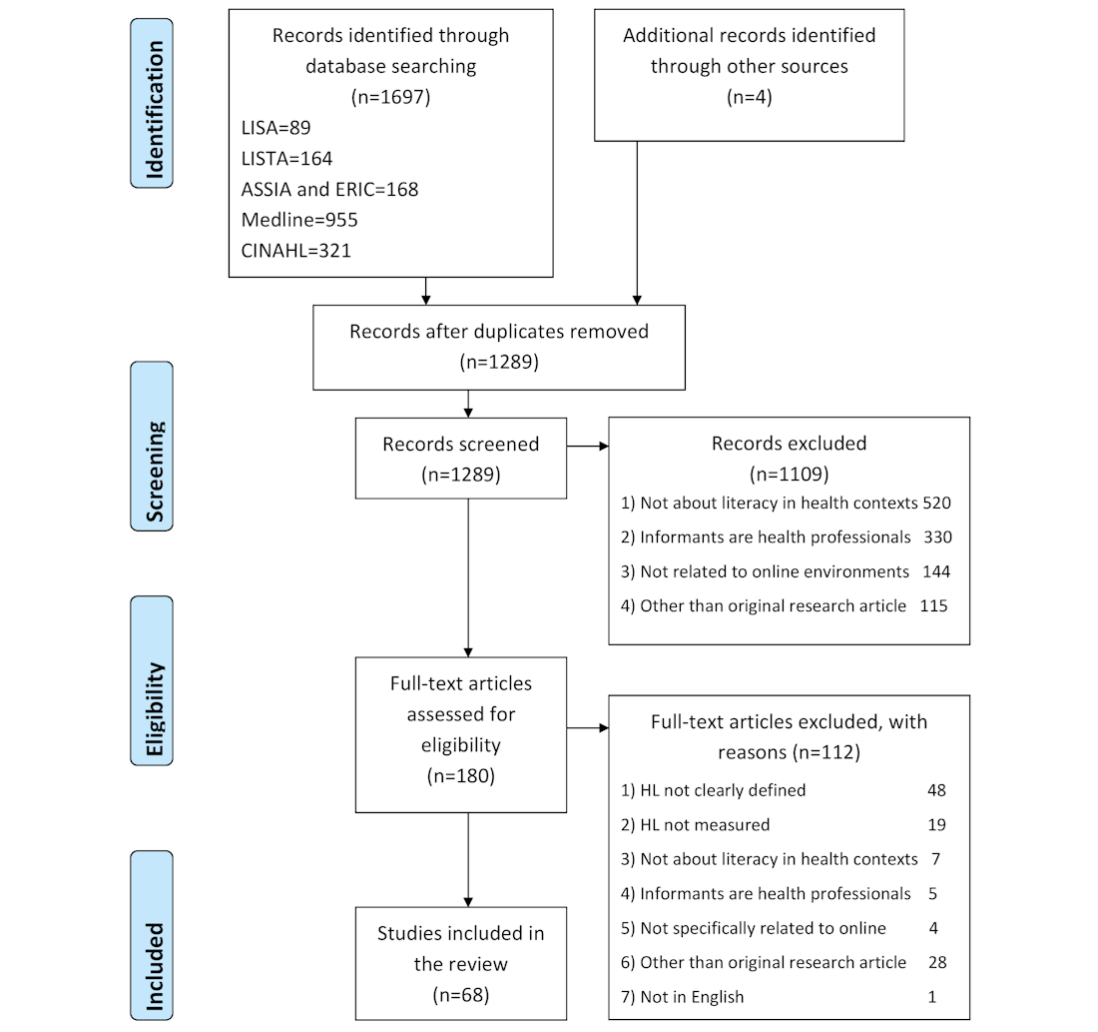
Preferred Reporting Items for Systematic Reviews and Meta-Analyses (PRISMA) flow diagram of the study selection process. LISA: Library and Information Science Abstracts; LISTA: Library and Information Science and Technology Abstracts; ASSIA: Applied Social Sciences Index and Abstracts; ERIC: Education Resources Information Center; CINAHL: Cumulative Index to Nursing and Allied Health Literature.

In addition, 1 academic journal (*Computers in Human Behavior*) was searched manually as it was not indexed in the searched databases but showed potential to finding relevant articles. Search from this journal was conducted by searching with the phrase “health” AND “literac*” OR “knowledge” from article titles and abstracts and within the same time frame as the database search. This search resulted in 4 relevant articles. In total, 1289 articles were identified through the literature search, as presented in Figure 1.

### Study Selection and Extraction of Data

The screening process of the articles was 2-phased. In the first phase, the duplicates were removed and the titles and abstracts of the articles (n=1289) screened independently by the first author to identify eligible articles for full-text screening. A 10% random sample was screened by the second author with an interrater agreement rate of 93%. The articles chosen for the full-text screening had to fulfill the following inclusion criteria: (1) original, peer-reviewed full-text article written in English; (2) the topic of the article concerned literacy in the context of health; (3) informants of the study were lay people, not health professionals or students of the field; and (4) the focus of study was health literacy in a Web-based information environment. In the second phase of the selection process, 180 full-text articles were screened, 112 of which were excluded.

After the study selection process, 68 articles were included in the review. The following data were extracted from these articles:

1. Title
2. Authors
3. Publication title
4. Year of publication
5. Research area or discipline (according to the first authors’ affiliation)
6. Aim or objective of the study
7. Method of data collection
8. Method of data analysis
9. Health literacy concept used
10. Definition of the concept
11. Measurement tool and its description.

A detailed description of the study selection process is presented in the PRISMA chart (see Figure 1). The characteristics of the included studies can be found in Multimedia Appendix 2.

## Results

### Characteristics of the Included Studies

In total, 68 studies were included in the systematic review. The studies represent a variety of disciplines (based on the first author’s affiliation), including medicine (n=13), health education and promotion or health communication (n=8), nursing (n=6), health sciences or public health (n=5), health policy (n=2), nutrition science (n=2), pharmacy (n=2), gerontology (n=1), biomedical informatics (n=1), communication or advertising (n=9), psychology (n=8), information science and information studies (n=8), sociology or social work (n=2), and behavioral sciences (n=1).

A total of 8 different health literacy concepts (Table 1) with 21 definitions (Multimedia Appendix 3) were identified. The most commonly used concepts were health literacy, which was referred to in 38 studies, and eHealth literacy, which was used in 37 studies. Other health-related literacy concepts that emerged were mental health literacy (n=3), oral health literacy (n=1), and *bad* health literacy (n=1). The concepts of health information literacy and everyday health information literacy were presented in 1 study. Refer to the study by Huhta et al [10] for a detailed description of the concepts and their definitions.

The most common method for data collection was a questionnaire survey, which was the only data collection method in 58 studies. There were 2 studies where interviews or focus groups were the only methods used. In 8 studies, several data collection methods were used. The analysis methods were predominantly quantitative (n=62). Mixed methods were applied in 4 studies and qualitative methods in 2 studies.

The included studies focused on different populations: patients or adults with risk factors for a disease (n=17), older adults or veterans (n=14), students (n=8), adults (n=8), and parents or caregivers (n=4). Other groups were participants with limited health literacy or computer literacy (n=2), middle-aged men (n=1), library users (n=1), members of an online support group (n=1), and the general public (n=12). The sample sizes ranged from 20 to 4368.

### Categorization

The content analysis focused on the health literacy concepts along with their definitions and measures. On the basis of the *definitions* of the health literacy concepts identified in the included articles, the studies were grouped into 3 thematic categories: health literacy as (1) a general skill, (2) a multidimensional concept, and (3) as a domain-specific concept. The categorization is drawn from the data, and it follows remarks made on health literacy research in earlier literature [9,25]. In Table 1, the identified definitions are presented in these categories.

If several concepts were cited, the main concept of the included study was derived from the article title, or if it was not mentioned, from the abstract. A detailed description of all identified concepts and their definitions is provided in Multimedia Appendix 3.

### Health Literacy as a General Skill

The definitions that describe health literacy as personal skills to utilize health information to gain better health were categorized as general and skill-based constructs. A general health literacy concept was adopted as the main concept in 23 studies. These studies referred to the health literacy definitions by Nutbeam [39], American Medical Association [40], Ratzan and Parker [41], Australian Bureau of Statistics [42], Rootman and Gordon-El-Bihbety [43], Berkman et al [44], The Patient Protection and Affordable Care Act [45], National Network of Libraries of Medicine [46], and the health information literacy definition by Shipman et al [20].

**Table 1:** Health literacy concepts identified in the included articles.

Thematic category	Concept	Defined by	Example of definition
General and skill-based	Health literacy; Health information literacy	Nutbeam [39], American Medical Association [40], Ratzan and Parker [41], Australian Bureau of Statistics [42], Rootman and Gordon-El-Bihbety [43], Berkman et al [44], The Patient Protection and Affordable Care Act [45], National Network of Libraries of Medicine [46]; Shipman et al [20]	Health literacy is “the degree to which individuals have the capacity to obtain, process, and understand basic health information and services needed to make appropriate health decisions.” Ratzan and Parker [41]
Multidimensional	Health literacy	Nutbeam [47], Zarcadoolas et al [48], Baker [49], Nutbeam [50], Sørensen et al [6]	“Health literacy is linked to literacy and entails people’s knowledge, motivation and competences to access, understand, appraise, and apply health information in order to make judgments and take decisions in everyday life concerning healthcare, disease prevention and health promotion to maintain or improve quality of life during the life course.” Sørensen et al [6]
Domain-specific	eHealth literacy; Mental health literacy; Oral health literacy; *Bad* health literacy	Norman and Skinner [15], Bodie and Dutta [51], Norman [52]; Jorm et al [53]; US Department of Health and Human Services [54]; Schultz and Nakamoto [55]	“eHealth literacy is defined as the ability to seek, find, understand, and appraise health information from electronic sources and apply the knowledge gained to addressing or solving a health problem.” Norman and Skinner [15]

The definition of health literacy as a capacity that individuals have in certain degrees by Ratzan and Parker [41] was cited in 24 studies [56-79]. Overall, it is the most often cited definition for health literacy in the included articles. Most of the articles cited a secondary source for the definition, such as that by the Healthy People 2010 initiative of the US Department of Health and Human Services [54]. The concept is process-oriented, focusing on obtaining basic health information and health services to make health decisions. A rather similar definition, but one with a wider scope including oral communication skills by Berkman et al [44] was cited in 5 studies [62,74,80-82]. This was the second most cited definition.

The health literacy definition adopted by the WHO and outlined by Nutbeam [39], stressing on both cognitive and social skills of an individual in the process of building motivation and understanding health information, was cited in 4 studies [83-86]. The health literacy definition by the American Medical Association [40] focused on individuals’ skills to perform tasks on reading comprehension and numeracy. It was cited in 2 studies [87,88]. Other definitions for general health literacy were cited only once and were rather similar to each other with only minor differences. For example, the definition by Rootman and Gordon-El-Bihbety [43] includes the attribute of evaluation and presents health literacy as an ability that can be improved across the life-course.

The concept of health information literacy by the Medical Library Association [20] presents individuals’ skills to recognize an information need, seek information, and use it as key competencies needed to make good health choices [20]. It was cited in 1 study [89]. In this definition, the focus is placed on the process of information seeking, described in more detail compared with the definitions for health literacy. The concept of health information literacy addresses also the individuals’ ability to assess the found information critically and to evaluate its applicability to a specific situation. This critical attribute is not present in all the definitions for health literacy and related concepts [10] and thus distinguishes the concept from other, more functional health literacy definitions.

Common for these definitions of health literacy and health information literacy is the focus on individuals’ abilities to obtain health information to make good health decisions. These definitions describe health literacy from 2 perspectives. First, health information is seen as general information obtained through information seeking. Second, health literacy is seen as a general skill set that an individual has to some degree and that it can be utilized universally in decision-making situations. Thus, health literacy is understood as a general, skill-based ability that can be applied to all kinds of situations that are related to health.

### Operationalization of the General Health Literacy Concepts

In total, 11 studies in this category used 1 or several measurement tools with an aim to detect the functional reading skills and numeracy of the selected population (see Table 2). The most often used functional measurement tools were the Newest Vital Sign [90] used in 4 studies [57,62,73,87] and the Rapid Estimate in Adult Literacy in Medicine (REALM) [91] used in 3 studies [56,58,63]. Other measurement tools used were The Test for Functional Health Literacy in Adults (TOHFLA) [21] (cited in [59]) and its shorter version S-TOFHLA [92] (cited in [84]), Short Assessment of Health Literacy in Dutch [93] (cited in [81]), and Adult Literacy & Life Skills Survey [94] (cited in [67]). These tools were developed to detect limited health literacy among adult patients in clinical settings.

Self-efficacy measures of health literacy were used in 5 studies [66,68,70,83,96] that adopted a general health literacy concept. Of these studies, 3 used a self-efficacy measure with only few screening items. Kim [66] states that individuals with higher levels of health literacy are expected to search health-related information from the Web more efficiently, and thus, in the study, health literacy was measured by asking whether the respondents searched for health information from the Web. Lee et al [68] used a 1-item health literacy screener by Chew et al [24], and Mayberry et al [70] used a modified 3-item version of the screener. It consists of questions about reading problems and confidence in filling out medical forms [24].

Other self-efficacy measures used were a reading comprehension screener called Single Item Literacy Screener [97] (cited in [71]) and the Functional Communicative and Critical Health Literacy scale (FCCHL) [23] (cited in [61]), which is based on Nutbeam’s [47] multidimensional definition of health literacy. FCCHL is a self-efficacy measure containing questions about the frequency of the patient’s actions, such as how often the patient had problems to read and comprehend medical texts (functional health literacy); how often they collect information, communicate about medical conditions, and apply the found information (communicative health literacy); and how often they critically evaluate the found information (critical health literacy) [23]. Furnival et al [89] used the Everyday Health Information Literacy (EHIL) screening tool by Niemelä et al [98] to measure the study participants’ health information literacy. The screening tool is based on the concept of health information literacy and was developed for studying “laypersons’ general and nonprofessional abilities related to health information” [98].

In addition, 2 studies [65,80] measured health literacy with a knowledge test. Jiang and Beaudoin [65] referred to Ratzan’s and Parker’s [41] definition of health literacy in their study and operationally defined the concept as “one’s knowledge and understanding on health-related issues.” The test consists of self-reported knowledge about medical research (scientific literacy), beliefs about US tobacco regulation (civic literacy), and a numeracy section. The authors suggested that the used knowledge test aligns with the multidimensional model of health literacy developed by Zarcadoolas et al [48]. Lee et al [80] cited the health literacy definition by Berkman et al [44] and stated that health knowledge is seen as a subdimension or a proxy of health literacy. In their study, health knowledge was measured by asking respondents to indicate the plausibility of 7 health statements [44]. Other types of measures identified were a skill-based health literacy performance test [74] and qualitative assessment of health-related information literacy [86].

**Table 2:** Operationalization of health literacy concepts in selected studies (N=68).

Type of measure	Thematic category, n (%)		
	General and skill-based (n=23)	Multidimensional (n=6)	Domain-specific (n=39)
Reading comprehension and numeracy	11 (16.2)	0 (0)	1 (1.5)
Self-efficacy	6 (8.8)	2 (2.9)	34 (50.0)
Knowledge	2 (2.9)	0 (0)	3 (4.4)
Performance tasks	1 (1.5)	0 (0)	0 (0)
Qualitative assessment	1 (1.5)	0 (0)	0 (0)
Several	2 (2.9)	4 (5.9)	1 (1.5)

Moreover, 2 studies [64,99] used several types of measures to assess general health literacy. In both studies, health literacy is defined as a skill-based construct, and it is assessed with reading comprehension and self-efficacy measures [64] or additionally also with a knowledge test [99]. For example, in a study by Woods et al [99], the study participants completed 11 different questionnaires that measured health knowledge, health literacy, and internet and computer skills. In 1 study [86], a qualitative assessment of health and information literacy was conducted.

Almost all the studies that adopted a general health literacy concept screen participants’ internet use [56,57,59,61-68,70, 71,74,77,80,84,86,87], usually with a simple yes or no question. In 4 studies [60,61,66,68] computer or internet literacy was measured, although in 2 of these, this means screening the internet use of the participants. In fewer cases, measures also included access to internet [59,63,87], skills [56,68,85,100] or comfort [70] to use internet or a computer, and abilities to communicate with peer or health professionals and providers in the Web [57,68]. In 3 studies [58,73,81] internet, computer, or technology-related measures were not included.

### Health Literacy as a Multidimensional Concept

Models that include several attributes, such as the social factors and cultural context into the definitions of health literacy, were categorized as multidimensional health literacy concepts. For example, the critical appraisal of found information is taken into account more thoroughly in these models. These multidimensional health literacy definitions and models by Nutbeam [47,50], Baker [49], Zarcadoolas et al [48], and Sørensen et al [6] were cited in 9 studies, the last 2 being the most used. In total, 6 studies chose the multidimensional construct as the main health literacy concept.

The health literacy definition by Zarcadoolas et al [48] was cited in 3 studies [72,83,88]. The definition includes the notion of health literacy as a lifelong learning process and sets the outcome of acquiring health literacy skills as an improved quality of life. This definition presents health and health literacy as the lifelong projects of *people*, not individuals. The model complementing the definition of health literacy by Zarcadoolas et al [48] is built around 4 central domains of literacy: fundamental, scientific, civic, and cultural. Of these, especially the domain of civic literacy represents the sociocultural aspect of literacy, as it includes “[u]nderstanding the relationship between one’s actions and the larger social group.” The civic literacy domain also stresses critical media literacy skills that include, for example, awareness of possible *biased authorities* in consumer advertising [48].

The health literacy definition by Sørensen et al [6] was cited in 3 studies [75,88,101]. Sørensen et al [6] reviewed health literacy research and created an integrated model with 6 dimensions of health literacy: (1) competence, skills, and abilities; (2) actions; (3) information and resources; (4) objective; (5) context; and (6) time. The definition considers individual capabilities, but it also aims to address the public health perspective [6].

Baker’s [49] conceptual model of health literacy was cited in 2 studies [102,103]. It presents several domains that affect health literacy. In the model, prior knowledge, such as vocabulary and conceptual knowledge of health together with reading fluency, is seen as a resource for an individual for facilitating health literacy. Health-related print and oral literacy are seen as dimensions of holistic health literacy that can lead to improved health outcomes. In addition, influencing factors, such as culture and norms, and barriers, such as limited access to health care, can have an effect on health behavior change [49].

Nutbeam [47] continued his examination on health literacy by broadening the definition into a conceptual model. The model consists of 3 literacy concepts: functional health literacy relates to health education and learning of factual information on health risks and on how to use the health system. Interactive health literacy concerns improving personal capacity to act independently on knowledge. Critical health literacy regards cognitive and skills development outcomes that support effective social and political action. According to Nutbeam [47], the first 2 literacy dimensions are effective on an individual level, but the third can also be seen linked to population level benefits. The model is developed to address the challenges for health education, and therefore, it presents health literacy as an outcome of health promotion. In his more recent article, Nutbeam [50] suggests that instead of conceptualizing health literacy as a risk factor influencing clinical outcomes, it should be seen as an asset that can support individual and population level health outcomes, when improved through patient education.

### Operationalization of the Multidimensional Health Literacy Concepts

In total, 6 studies [75,83,88,101,103,108] adopted a multidimensional health literacy concept as the central concept of the study. The operationalization of these concepts varied, and several types of measures were used, as seen in Table 2.

Rowsell et al [101] referred to the multidimensional health literacy definitions by Sørensen et al [6] and Nutbeam [50] and evaluated the level of health literacy with a single-item self-efficacy measure by Chew et al [24] with the aim to detect patients’ difficulties in understanding written information. On the other hand, van der Vaart et al [103] adopted Baker’s [49] health literacy definition as their main literacy concept and measured it with the FCCHL self-efficacy scale that includes several literacy domains.

In 4 studies, several types of measures were used. In a study by Tam et al [75], the combination of measures included a reading comprehension and numeracy measure the Rapid Estimate of Adult Literacy in Medicine and Dentistry measure (REALMD-20) [107], a 2-item self-efficacy measure by Chew et al [24], and a dental health knowledge test. In this study, oral health literacy was measured, although the authors did not provide a clear definition of the concept itself. Instead, the health literacy definition by Sorensen et al [6] and the concept of eHealth literacy [15] were discussed. In other studies that adopted a multidimensional health literacy concept, reading comprehension and numeracy [88], self-efficacy [83,88,108], knowledge [88,108], and performance [83] were measured.

Computer or internet literacy was not measured in studies that adopted a multidimensional concept of health literacy. Instead, internet access [103,108] and use [103] were screened. Subramania et al [88] included internet-related questions to their overall assessment of health literacy skills of the participants. Moreover, 3 studies [75,83,101] did not include any kinds of internet- or computer-related measures to their study.

### Health Literacy as a Domain-Specific Concept

The health literacy concepts that focus on a specific context or target a specific patient group are categorized as domain-specific concepts of health literacy. In total, a domain-specific concept of health literacy was cited in 41 of the included studies. Of these, eHealth literacy by Norman and Skinner [15], Bodie and Dutta’s [51] elaboration of the same concept, and Norman’s [52] suggestion of eHealth literacy 2.0 definition are essentially targeted to address health literacy in Web contexts. Of these, Norman’s and Skinner’s definition was the most often cited definition in included studies. In several studies (n=11), in addition to eHealth literacy, also other health literacy concepts and definitions were discussed (see Multimedia Appendix 2).

In total, 39 studies adopted a domain-specific health literacy concept as the main concept of the study. In most of these studies (n=34), the main concept was eHealth literacy [60,69,72,76-79,82,85,95,96,100-102,104-106,109-126]. The concept of eHealth literacy is accompanied by the Lily model that consists of 6 literacies organized in 2 central types: analytic (traditional, media, and information) and context-specific (computer, scientific, and health). The analytic literacy types are described as skills that are applicable to a wide range of information sources [15]. The context-specific types involve skills that are applied in specific situations. According to Norman and Skinner [15], all these skills are required when engaging with electronic sources. In the definition of eHealth literacy, the electronic element of health information seeking seems to be addressed as a contrast to nonelectronic information seeking, although a deeper explanation of those electronic sources is absent in the definition [15].

Bodie and Dutta [51] present an elaborated definition for eHealth literacy that stresses the significance of the Web context in seeking, evaluating, and using health information. This definition was presented in 1 study [114]. Norman’s [52] definition for eHealth literacy 2.0 was presented in 1 study [126]. With the definition, Norman attempts to emphasize the context of social media regarding eHealth literacy screening tool development by presenting social media relevant tasks and skills to the concept [52].

Other domain-specific health literacy concepts identified in the studies were mental health literacy used in 3 studies [127-129], oral health literacy used in 1 study [130], and *bad* health literacy used in 1 study [31]. The definition of mental health literacy by Jorm et al [53], unlike other health literacy definitions, also addresses beliefs and attitudes toward health issues. The definition of oral health literacy by the US Department of Health and Human Services [54] is based on the health literacy definition by Ratzan and Parker [41] and thus takes a skill-based approach to the concept. The concept of *bad* health literacy originally introduced by Schulz and Nakamoto [55] refers, according to Allam et al [131], to “the presence of the ability to understand medical information turned sour by the simultaneous absence of the ability to recognize it as false.” In other words, the information seeker might be literate enough to find, understand, and process even low-quality information, obtained, for example, from electronic sources but is incapable to recognize it as false, irrelevant, or fraudulent [131].

### Operationalization of the Domain-Specific Health Literacy Concepts

Within the studies that adopted a domain-specific concept as the main health literacy concept (n=39), the operationalization is more often done with a self-efficacy measurement tool than other types of measures, as seen in Table 2.

Most of the studies that adopted eHealth literacy as the main concept used the eHealth Literacy Scale (eHEALS) by Norman and Skinner [132] as the main measurement tool. In total, the eHEALS is used in 29 of the 39 studies in this category and as the only used tool in 25 of them [60,72,76,77,79,82,85, 95,96,100,102,104-106,113,115-123,125]. The 8-item eHEALS scale aims to measure “consumers’ combined knowledge, comfort, and perceived skills at finding, evaluating, and applying electronic health information to health problems.” The scale is proposed to address the 6 literacy types of the Lily model [15]. In the included studies, the eHEALS is described in different ways. Typically, the scale is described as a measurement that detects consumers’ perceived information technology or computer skills. In addition, the abilities to seek health information from the Web are seen as central attributes of the scale. Other studies that adopted the eHealth literacy as the main concept of the study also used other self-efficacy measures, such as EHIL [98] (cited in [110]) and Brief Health Literacy Screening Tool BRIEF [133] (cited in [112]). In addition, 2 studies [69,114] present a new eHealth literacy measure. Hsu et al [114] discuss eHealth literacy definitions by Norman and Skinner [15] and Bodie and Dutta [51] and present a new eHealth literacy measure eHL that seeks to detect individuals’ “ability to seek, find, understand, and evaluate health information from electronic sources and apply this knowledge to address or solve a health problem” [114]. The self-efficacy measure eSEARCH, eHealth Literacy Tool used in a study by Manafò et al [69], was developed to measure eHealth literacy skills of older adults.

Other types of measures used in the included articles that adopted eHealth literacy as the main concept were performance tests [109,126]; combined measures of reading comprehension, numeracy, and knowledge [124]; and self-efficacy [78]. In 1 study [111], eHealth literacy was assessed qualitatively based on focus group discussions of the participants.

Mental health literacy was measured in 3 studies [127-129] and oral health literacy [130] and *bad* health literacy [131] both in 1 study. In 2 of the studies that focused on mental health literacy [128,129], the concept was operationalized by measuring the participants’ knowledge about and attitudes toward mental health issues. Li et al [127] used several types of measures. The 31-item questionnaire consists of questions about the participant’s knowledge and self-efficacy on mental health issues. In a study by Tse et al [130], oral health literacy was measured with REALD-30 [134], a word recognition instrument that requires participants to read aloud 30 oral health–related words. Allam et al [131] measured *bad* health literacy with a knowledge test focused on vaccine information.

## Discussion

### Principal Findings

The aim of this systematic review was to identify health literacy concepts and their definitions and operationalization in studies focused on Web-based information environments. The concept of eHealth literacy by Norman and Skinner [15] was used most often. However, the concept of health literacy was also used and a variety of definitions were presented for it in the selected studies. On the basis of the definitions for health literacy, 3 thematic categories were identified, namely, general and skill-based, multidimensional, and domain-specific. Most studies adopted a domain-specific concept, followed by the ones that used a general and skill-based concept. Multidimensional concepts occurred least frequently.

The general concept of health literacy was typically operationalized by using reading comprehension and numeracy measures. In turn, the domain-specific concepts were most often operationalized by using a self-efficacy measure. Several types of measures were used in studies that adopted multidimensional constructs of health literacy. Nevertheless, inconsistencies in the operationalization of the different concepts were identified.

### Comparison With Prior Work

The lack of consensus in defining health literacy, as presented in several reviews [6,11,30], is supported by the results of this systematic review as several different definitions for the concept were identified in the included studies. The modern health literacy definitions are more often multidimensional than functional [3,9]. However, this systematic review shows that there is a tendency to refer to the early definitions of health literacy, which present a functional understanding of the concept. Within the studies that applied the concept of eHealth literacy, a more consistent understanding of the definition was detected as only 2 definitions for the concept were presented.

As earlier reviews indicate, the currently used measures of health literacy have focused on assessing individuals’ reading comprehension and understanding of medical texts in clinical contexts [5,135]. In addition, within the studies conducted in Web-based information environments, general health literacy was measured with a widely used and validated functional measurement tool, although there are more recent and multidimensional measures available [25]. Pleasant et al [135] argue that the focus on measuring only the functional skills of individuals leaves important factors such as individual information and communication skills untested. Despite the trend of understanding health literacy as a multidimensional construct including contextual, cultural, and social factors [5], these were not acknowledged in the studies included in this systematic review.

The concept of eHealth literacy by Norman and Skinner [15] was clearly the most used concept in the included studies. As a domain-specific concept, eHealth literacy aims to address especially the literacy skills needed in Web contexts. However, in the included studies, the concept was described as the technological skills of the study subjects. Yet, it is clear that eHealth literacy competencies are more varied than the mere ability to use the internet or a computer efficiently. Addressing literacy skills or practices through domain-specific concepts offers an opportunity to express domain-specific issues, such as the importance of the technological skills as part of eHealth literacy competencies, or oral health knowledge as part of oral health literacy. However, the development of these concepts may be challenging, as the focus of research is fragmented in empirical studies and the conceptual development is scarce (See also [8]).

Measurement of eHealth literacy is more often focused on assessing the self-reported skills of individuals. Unlike in the systematic review by Karnoe and Kayser [28], dual-design eHealth literacy measures are not common in studies conducted in Web-based information environments, as only few studies included internet or digital literacy measures in their health literacy screening tools.

The trend toward mixing different measuring types, as indicated by Altin et al [25], was noted also within the studies conducted in Web-based information environments. The focus on clinical settings as a study context was not as clearly indicated as in the earlier reviews, and usually, the sample population was a certain age instead of patients.

### Strengths and Limitations

To our knowledge, this study is among the first cross-disciplinary reviews of health literacy concepts, definitions, and their operationalization in Web contexts. The systematic process of this review enabled thorough investigation of the health literacy–related academic research focused on the context of Web-based information environments. The main limitations of this review lie within the search strategy. Only studies written in English were included in the review, which excluded relevant studies in other languages. In addition, some studies may have been missed due to the restricted search terms and limited time frame.

### Conclusions

This systematic review identified health literacy concepts, definitions, and operationalization used in research focusing on Web-based information environments. On the basis of the results, several concepts are being used, eHealth literacy and health literacy being the most common ones. In addition, 3 thematic categories of the different definitions were identified: general and skill-based, multidimensional, and domain-specific. Typically, general and skill-based health literacy was measured with reading comprehension or numeracy tests and domain-specific health literacy with self-efficacy tests. Multidimensional concepts were used less often and operationalized by using several types of measures. Future studies conducted in Web contexts should place emphasis on the conceptual development of health literacy. Researchers are encouraged to provide clear operationalization for the concepts they use to ensure transparency in reporting.

## Appendix

Multimedia Appendix 1 Search strategy.

Multimedia Appendix 2 Characteristics of the included studies.

Multimedia Appendix 3 Health literacy concepts and definitions in the included studies.

## Abbreviations

BRIEF: Brief Health Literacy Screening Tool
eSEARCH: eHealth Literacy Tool
eHealth: electronic health
eHEALS: eHealth Literacy Scale
EHIL: Everyday Health Information Literacy
FCCHL: Functional Communicative and Critical Health Literacy scale
PRISMA: Preferred Reporting Items for Systematic Reviews and Meta-Analyses
REALM: Rapid Estimate in Adult Literacy in Medicine
TOHFLA: Test for Functional Health Literacy in Adults
WHO: World Health Organization
