# Supplementary material for: Health Literacy in Web-Based Health Information Environments: Systematic Review of Concepts, Definitions, and Operationalization for Measurement
Source: J Med Internet Res. 2018 Dec 19;20(12):e10273. doi: 10.2196/10273 (PMC6315258; doi:10.2196/10273)
Supplement: Multimedia Appendix 1 [file jmir_v20i12e10273_app1.pdf]

Multimedia Appendix 1. The search strategy.

Online OR internet OR digital OR web OR "social media" OR SNS* OR "social networking site*" OR "social networking service*" OR facebook OR twitter OR youtube OR instagram OR snapchat OR periscope<br><br>AND<br><br>health* OR wellbeing OR well-being OR wellness OR disease OR health OR e-health OR ehealth<br><br>AND<br><br>literacy OR literacies OR comprehend* OR "abilit* N2 (read* OR search* OR seek* OR writ* OR evaluat* OR assess*)" OR "comprehen* N2 read*" OR "skill* N2 (read* OR search* OR seek* OR writ* OR evaluat* OR assess*)" OR competenc* OR "health knowledge" OR numeracy
