# Supplementary material for: Health Literacy in Web-Based Health Information Environments: Systematic Review of Concepts, Definitions, and Operationalization for Measurement
Source: J Med Internet Res. 2018 Dec 19;20(12):e10273. doi: 10.2196/10273 (PMC6315258; doi:10.2196/10273)
Supplement: Multimedia Appendix 2 [file jmir_v20i12e10273_app2.pdf]

Multimedia Appendix 2. Characteristics of the included studies (N=68).

**Study and year**	**Research area**	**Method of data collection to study health literacy**	**Method of data analysis**	**Sample and target population**	**Health literacy concept used (main concept first), defined by**	**Tool used to assess health literacy**
Agree et al, 2015 [56]	Sociology	Questionnaire survey	Quantitative	346 adults over 35 years	Health literacy by Ratzan and Parker [41]	REALM[a]
Allam et al, 2015 [131]	Communication	Questionnaire survey performance tests	Quantitative	39 marketing students + 197 adults	*Bad* Health literacy by Schulz and Nakamoto [55]	knowledge test (vaccination)
Austvoll-Dahlgren et al, 2012 [83]	Health sciences	Questionnaire survey, task data	Quantitative	96 parents	Health literacy by Nutbeam [39,47]; Zarcadoolas et al [48]	Search tasks and DISCERN[b], TPB[c], PAM[d]
Bailey et al, 2015 [57]	Pharmacy	Questionnaire survey	Quantitative	1077 patients	Health literacy by Ratzan and Parker [41]	NVS[e]
Bickmore et al, 2016 [58]	Computer and information science	Questionnaire survey	Quantitative	89 participants with low HL and computer literacy	Health literacy by Ratzan and Parker [41]	REALM
Blackstock et al, 2016 [104]	Medicine	Interviews	Quantitative	63 women with HIV	eHealth literacy by Norman and Skinner [15]	eHEALS[f]
Chan et al, 2011 [109]	Biomedical informatics	Performance tasks, audio-recordings	Mixed	1 user in pilot phase, 20 users in testing	eHealth literacy by Norman and Skinner [15]	Cognitive task analysis + perfomance tasks
Chang et al, 2015 [110]	Health education	Questionnaire survey	Quantitative	1869 students and 1365 parents	eHealth literacy by Norman and Skinner [15]	eHEALS; EHIL[g]
Chisolm et al, 2011 [59]	Medicine	Questionnaire survey	Quantitative	180 adolescents	Health literacy by Ratzan and Parker [41]	TOFHLA[h]
Choi et al, 2013 [100]	Gerontology	Questionnaire survey	Quantitative	270 under 60 yrs, 763 over 60 yrs	eHealth literacy by Norman and Skinner [15]	eHEALS
Chung et al, 2015 [60]	Nursing	Questionnaire survey	Quantitative	866 older adults	eHealth literacy by Norman and Skinner [15] Health literacy by Ratzan and Parker [41]	eHEALS
Cnossen et al, 2016 [61]	Medicine	Questionnaire survey	Quantitative	38 patients	Health literacy by Ratzan and Parker [41]	FCCHL (Dutch)[i]
Connolly et al, 2015 [111]	Medicine	Focus groups	Qualitative	25 adults with diabetes	eHealth literacy by Norman and Skinner [15]	Qualitative assessment
Crook et al, 2016 [62]	Communication	Questionnaire survey	Quantitative	180 adults from health care clinic	Health literacy by Berkman et al [44]; Ratzan and Parker [41]	NVS
Duncan et al, 2015 [108]	Medicine and public health	Questionnaire survey	Quantitative	301 middle-aged men	Health literacy by Nutbeam [50]; Australian Bureau of Statistics [42]	Active Australia Questionnaire + Dietary behavior literacy
Echt et al, 2011 [84]	Medicine	Questionnaire survey	Quantitative	250 older adults	Health literacy by Nutbeam [39]	S-TOFHLA[j]

Fagnano et al, 2012 [63]	Medicine	Questionnaire survey	Quantitative	304 caregivers of children with astma	Health literacy by Ratzan and Parker [41]	REALM
Furnival et al, 2015 [89]	Information science	Questionnaire survey, interviews	Quantitative	73 participants	Health Information Literacy by Shipman et al [20]	EHIL (adapted)
Ghaddar et al, 2012 [64]	Health promotion, public health	Questionnaire survey	Quantitative	261 students	Health literacy by Ratzan and Parker [41]	eHEALS, NVS
Gutierrez et al, 2015 [87]	Medicine	Questionnaire survey	Quantitative	498 adult patients	Health literacy by the American Medical Association [40]	NVS + confidence in filling medical forms
Haun et al, 2015 [112]	Health communication and promotion	Questionnaire survey	Quantitative	819 US veterans	eHealth literacy by Norman and Skinner [15]	BRIEF[k], eHEALS
Hogan et al, 2016 [113]	Medicine	Questionnaire survey	Quantitative	290 US veterans	eHealth literacy by Norman and Skinner [15]	eHEALS
Hsu et al, 2015 [114]	Health education	Questionnaire survey	Quantitative	525 college students	eHealth literacy by Norman and Skinner [15]; Bodie and Dutta [51]	eHLS[l]
Hu et al, 2012 [95]	Communication	Questionnaire survey	Quantitative	505 patients and members of an online support group	eHealth literacy by Norman and Skinner [15]	eHEALS
Jiang et al, 2016 [65]	Communication	Questionnaire survey	Quantitative	2305 participants	Health literacy by Ratzan and Parker [41]	HINTS[m] health literacy screening measure (adapted)
Kim, 2013 [66]	Information studies	Questionnaire survey	Quantitative	3002 observations	Health literacy by Ratzan and Parker [41]	Single-item: health information search online (yes/no)
Knapp et al, 2011 [115]	Health policy	Questionnaire survey	Quantitative	129 parents	eHealth literacy by Norman and Skinner [15]	eHEALS
Knapp et al, 2011 [116]	Health policy	Questionnaire survey	Quantitative	2371 parents	eHealth literacy by Norman and Skinner [15]	eHEALS
Kobayashi et al, 2015 [67]	Epidemiology and public health	Questionnaire survey	Quantitative	4368 older adults	Health literacy by Ratzan and Parker [41]	Adult Literacy & Life Skills Survey
Koo et al, 2012 [117]	Health sciences	Questionnaire survey	Quantitative	219 sixth-grade school children	eHealth literacy by Norman and Skinner [15]	eHEALS
Lee et al, 2012 [80]	Communication	Questionnaire survey	Quantitative	1812 adults	Health literacy by Berkman et al [44]	Seven health knowledge items
Lee et al, 2015 [96]	Pharmacy	Questionnaire survey	Quantitative	400 patients	eHealth literacy by Norman and Skinner [15]	eHEALS
Lee et al, 2015 [68]	Nursing	Questionnaire survey	Quantitative	2680 hispanic adults	Health literacy by Ratzan and Parker [41]	One-item screening instrument by Chew et al

Li et al, 2015 [118]	Communication	Questionnaire survey	Quantitative	311 members of online support group	eHealth literacy by Norman and Skinner [15]	eHEALS
Li et al, 2013 [127]	Social work and social adminstration	Questionnaire survey	Quantitative	221 undergraduate students	Mental health literacy by Jorm et al [53]	31 questions; understanding of mental health concepts and application of skills
Lintvedt et al, 2013 [128]	Psychology	Questionnaire survey	Quantitative	163 young adults at risk of depression	Mental health literacy by Jorm et al [53]	Treatment depression literacy; knowledge based
Manafò et al, 2012 [85]	Nutrition science	Questionnaire survey	Mixed	48 older adults	eHealth literacy by Norman and Skinner [15] Health literacy by Nutbeam [39]	eHEALS (adapted)
Manafò et al, 2013 [69]	Nutrition science	Questionnaire survey	Quantitative	67 older adults	eHealth literacy by Norman and Skinner [15] Health literacy by Ratzan and Parker [41]; Rootman and Gordon-El-Bihbety [43]	eHEALS; eSEARCH[n]
Marshall, et al, 2012 [86]	Information science	Questionnaire survey, workshops, interviews	Qualitative	385 answered to survey, 39 participated in the workshop, 18 interviewed	Health literacy by Nutbeam [39] eHealth literacy by Norman and Skinner [15]	Qualitative assessment of information literacy regarding health
Mayberry et al, 2011 [70]	Medicine	Questionnaire survey	Quantitative	61 adults with type 2 diabetes	Health literacy by Ratzan and Parker [41]	Three-item HL screening instrument by Chew (modified)
McCleary-Jones et al, 2013 [71]	Nursing	Questionnaire survey	Quantitative	88 African-American adults	Health literacy by Ratzan and Parker [41]; the American Medical Association [40]	SILS[o]
Meppelink et al, 2015 [81]	Communication	Questionnaire survey, audio-recordings	Quantitative	61 participants with low or limited HL	Health literacy by Berkman et al [44]	Short Assessment of Health Literacy in Dutch (SAHL-D)
Milne et al, 2015 [105]	Medicine	Questionnaire survey	Quantitative	83 cancer patients	eHealth literacy by Norman and Skinner [15]	eHEALS
Mitsutake et al, 2012 [119]	Health and Behavioral Sciences	Questionnaire survey	Quantitative	2970 Japanese adults	eHealth literacy by Norman and Skinner [15]	eHEALS (Japanese version)
Nahm et al, 2012 [120]	Nursing	Questionnaire survey	Quantitative	27 caregiver-care receiver dyads (pair)	eHealth literacy by Norman and Skinner [15]	eHEALS
Neter et al, 2012 [106]	Behavioral sciences	Questionnaire survey	Quantitative	4286 Israeli adults	eHealth literacy by Norman and Skinner [15]	eHEALS
Ossebaard et al, 2012 [121]	Public health	Questionnaire survey	Quantitative	21 patients	eHealth literacy by Norman and Skinner [15]	eHEALS (Dutch version)
Paek et al, 2012 [72]	Advertising and public relations	Questionnaire survey	Quantitative	182 students	eHealth literacy by Norman and Skinner [15] Health literacy by Ratzan and Parker [41]; Zarcadoolas, Pleasant and Greer [48]	eHEALS
Park et al, 2016 [122]	Nursing	Questionnaire survey	Quantitative	108 library users	eHealth literacy by Norman and Skinner [15]	eHEALS

Park et al, 2016 [123]	Nursing	Questionnaire survey	Quantitative	108 adults	eHealth literacy by Norman and Skinner [15]	eHEALS
Reininger et al, 2013 [124]	Health promotion and behavioral sciences	Questionnaire survey	Quantitative	71 adults at risk for diabetes	eHealth literacy by Norman and Skinner [15]	TOFHLA; diabetes knowledge
Rowsell et al, 2015 [101]	Psychology, medicine	Questionnaire survey	Quantitative	64 adults	Health literacy by Nutbeam [50]; Sørensen et al [6]	One-item screening instrument by Chew et al + HLS-EU-16[p]
Sheng et al, 2013 [125]	Psychology	Questionnaire survey	Quantitative	771 older adults	eHealth literacy by Norman and Skinner [15]	eHEALS
Smith et al, 2015 [73]	Medicine	Questionnaire survey	Quantitative	534 older adults	Health literacy by Ratzan and Parker [41]	NVS
Subramaniam et al, 2015 [88]	Information studies	Questionnaire survey, observation, interviews, focus groups, web portal data	Mixed	30 students	Health literacy by Sørensen et al [6]; Zarcadoolas, Pleasant and Greer [48]; National Network of Libraries of Medicine [46]; the American Medical Association [40]	Health literacy skills inventory (a literature based design)
Sun et al, 2013 [74]	Social medicine and health education	Questionnaire survey	Quantitative	3222 participants	Health literacy by Ratzan and Parker [41]; Berkman et al [44]	A skills-based HL intstrument regarding infectious respiratory diseases (Sun)
Tam et al, 2015 [75]	Medicine (dentistry)	Questionnaire survey	Quantitative	100 adults	Health literacy by Sørensen et al [6]; Ratzan and Parker [41]<br>eHealth literacy by Norman and Skinner [15]	REALM-D[q]; dental knowledge, a two-item HL screening instrument by Chew et al
Taylor-Rodgers et al, 2015 [129]	Psychology	Questionnaire survey	Quantitative	56 young adults	Mental health literacy by Jorm et al [53]	Literacy of Suicide Scale, A-lit[r], D-lit
Tennant et al, 2015 [76]	Health education and promotion	Questionnaire survey	Quantitative	283 baby boomers and older adults	eHealth literacy by Norman and Skinner [15]<br>Health literacy by Ratzan and Parker [41]	eHEALS
Tse et al, 2015 [130]	Health education	Questionnaire survey	Quantitative	22 adolescents	Oral health literacy by the U.S. Department of Health and Human Services [54]	REALD-30[t]
van der Vaart et al, 2011 [102]	Psychology	Questionnaire survey	Quantitative	189 patients with rheumatic diseases (study 1), 88 Dutch adults (study 2)	eHealth literacy by Norman and Skinner [15]<br>Health literacy by Baker [49]	eHEALS
van der Vaart et al, 2013 [126]	Psychology	Performance tasks, think-aloud method, interview	Mixed	15 + 16 patients	eHealth literacy by Norman and Skinner [15]; eHealth literacy 2.0 by Norman [52]	Performance tasks
van der Vaart et al, 2011 [103]	Psychology	Questionnaire survey	Quantitative	227 patients	Health literacy by Baker [49]	FCCHL (Dutch version)

Wenhong et al, 2015 [82]	Communication	Questionnaire survey	Quantitative	540 students	eHealth literacy by Norman and Skinner [15] Health literacy Berkman et al [44]	eHEALS
Woods et al, 2016 [99]	Psychology	Questionnaire survey	Quantitative	46 participants with HIV-infection	Health literacy by The Patient Protection and Affordable Care Act [45]	eHEALS, REALM, HIV Knowledge 18, Expanded Numeracy Scale, Short Assessment of Health Literacy, TOFHLA, NVS
Xie, 2011 [77]	Information studies	Questionnaire survey	Quantitative	172 older adults	eHealth literacy by Norman and Skinner [15] Health literacy by Ratzan and Parker [41]	eHEALS
Xie, 2012 [78]	Information studies	Questionnaire survey	Quantitative	218 older adults	eHealth literacy by Norman and Skinner [15] Health literacy by Ratzan and Parker [41]	Computer Anxiety Scale; Attitudes Toward Computers Questionnaire (Efficacy and interest subscales); Computer and Web knowledge tests; Questionnaire of the "satisfaction with the training and changes in participation in one's own health care"
Xie, 2011 [79]	Information studies	Questionnaire survey	Quantitative	156 older adults	eHealth literacy by Norman and Skinner [15] Health literacy by Ratzan and Parker [41]	eHEALS

a Rapid Estimate of Adult Literacy in Medicine

b DISCERN: an instrument for judging the quality of written consumer health information on treatment choices.

c Theory of Planned Behavior

d Patient Activation Measure

e Newest Vital Sign

f eHealth Literacy Scale

g Everyday Health Information Literacy Screening Tool

h Test for Functional Health Literacy in Adults

i Functional, Communicative and Critical Health Literacy Scale

j Short Test for Functional Health Literacy in Adults

k Brief Health Literacy Screening Tool

l eHealth Literacy Scale

m Health Information National Trends Survey

n eSEARCH eHealth Literacy Tool

o Single Item Literacy Screener

p Health Literacy Survey – Europe – 16 Questions

q Rapid Estimate of Adult Literacy in Medicine and Dentistry

r Anxiety Literacy Questionnaire

s Depression Literacy Questionnaire

t Rapid Estimate of Adult Literacy in Dentistry
