# Supplementary material for: Health Literacy in Web-Based Health Information Environments: Systematic Review of Concepts, Definitions, and Operationalization for Measurement
Source: J Med Internet Res. 2018 Dec 19;20(12):e10273. doi: 10.2196/10273 (PMC6315258; doi:10.2196/10273)
Supplement: Multimedia Appendix 3 [file jmir_v20i12e10273_app3.pdf]

Multimedia Appendix 3. Health literacy concepts and definitions in the included articles.

Thematic group and concept	Defined by, year	Definition	Cited in
**General and skills-based**			
Health literacy	Nutbeam, 1998 [39]	“[T]he cognitive and social skills which determine the motivation and ability of individuals to gain access to, understand and use information in ways which promote and maintain good health.”	[83-86]
	American Medical Association, 1999 [40]	“[C]onstellation of skills, including the ability to perform basic reading and numerical tasks required to function in the health care environment.”	[87,88]
	Ratzan and Parker, 2000 [41]	"[T]he degree to which individuals have the capacity to obtain, process, and understand basic health information and services needed to make appropriate health decisions."	[56-79]
	Australian Bureau of Statistics, 2006 [42]	“[T]he knowledge and skills required to understand and use information relating to health issues such as drugs and alcohol, disease prevention and treatment, safety and accident prevention, first aid, emergencies, and staying healthy.”	[108]
	Rootman and Gordon-El-Bihbety, 2006 [43]	“The ability to access, understand, evaluate and communicate information as a way to promote, maintain and improve health in a variety of settings across the life-course.”	[80]
	Berkman et al, 2010 [44]	“The degree to which individuals can obtain, process, understand, and communicate about health-related information needed to make informed health decisions.”	[74,80-82,95]
	The Patient Protection and Affordable Care Act, 2010 [45]	“The term ‘health literacy’ means the degree to which an individual has the capacity to obtain, communicate, process, and understand health information and services in order to make appropriate health decisions.”	[99]
	National Network of Libraries of Medicine, 2013 [46]	“Health literacy requires a complex group of reading, listening, analytical, and decision-making skills, as well as the ability to apply these skills to health situations.”	[88]
Health information literacy	Shipman et al, 2009 [20]	“[T]he set of abilities needed to: recognize a health information need; identify likely information sources and use them to retrieve relevant information; assess the quality of the information and its applicability to a specific situation; and analyze, understand, and use the information to make good health decisions.”	[89]

**Multidimensional**			
Health literacy	Nutbeam, 2000 [47]	"Health literacy refers to the personal, cognitive and social skills which determine the ability of individuals to gain access to, understand, and use information to promote and maintain good health."; "Basic/functional literacy", "Communicative/interactive literacy", "Critical literacy".	[83]
	Baker, 2006 [48]	Conceptual model of the relationship between individual capacities, health-related print and oral literacy, and health outcomes: Two subdomains: 1) reading fluency and prior knowledge (vocabulary and conceptual knowledge of health and health care) 2) health literacy (health related print and oral literacy). Other factors that influence: Culture and norms, barriers to change.	[102,103]
	Zarcadoolas et al, 2006 [49]	"… wide range of skills and competencies that people develop over their lifetimes to seek out, comprehend, evaluate, and use health information and concepts to make informed choices, reduce health risks, and increase quality of life."	[118,120,123]
	Nutbeam, 2008 [50]	Conceptual model of health literacy as an asset: "it commences with recognition of prior knowledge and capability, leading to tailored health education and communication. -- People who have better developed health literacy will thus have skills and capabilities that enable them to engage in a range of health enhancing actions including personal behaviours, as well as social actions for health and the capability of influencing others towards healthy decisions such as quitting smoking, or participating in preventative screening programs."	[40,103]
	Sørensen et al, 2012 [6]	"Health literacy is linked to literacy and entails people's knowledge, motivation and competences to access, understand, appraise, and apply health information in order to make judgments and take decisions in everyday life concerning healthcare, disease prevention and health promotion to maintain or improve quality of life during the life course."	[75,88,101]
**Domain-specific**			
eHealth literacy	Norman and Skinner, 2006 [15]	"eHealth literacy is defined as the ability to seek, find, understand, and appraise health information from electronic sources and apply the knowledge gained to addressing or solving a health problem."	[60,69,72,75-79,82,85,86,95,96,100,102,104-106,109,110-126]
	Bodie and Dutta, 2008 [51]	"Thus, high eHealth literacy is not just the ability to use the Internet to find answers to health-related questions (e.g., devise appropriate search strategies, find information on poorly mapped sites); it also entails the ability to understand the information found (e.g.. What does it mean? What does it mean for me?), evaluate the veracity of this information (e.g., Can I trust this source? Does the information found from multiple sites conflict or agree?), discern the quality of different health Web sites (e.g. Is this site sponsored by associations with potential conflicts of interest?), and use quality information to make informed decisions about health."	[114]

eHealth literacy 2.0.	Norman, 2011 [52]	"Items could be developed that consider skills and tasks like:<br>• confidence in expressing oneself clearly in social interactions online<br>• ability to synthesize professional and non-professional advice<br>• comfort and skill in navigating through information obtained through a mobile device<br>• ability to use apomediaries to filter relevant and trustworthy information"	[126]
Mental health literacy	Jorm et al, 1997 [53]	"[K]nowledge and beliefs about mental disorders which aid their recognition, management or prevention". "Mental health literacy includes the ability to recognise specific disorders; knowing how to seek mental health information; knowledge of risk factors and causes, of self-treatments, and of professional help available; and attitudes that promote recognition and appropriate help-seeking."	[127-129]
Oral health literacy	U.S. Department of Health and Human Services, 2000 [54]	"Based on the definition of health literacy, the degree to which individuals have the capacity to obtain, process, and understand basic oral and craniofacial health information and services needed to make appropriate health decisions."	[130]
Bad health literacy	Schulz and Nakamoto, 2011 [55]	"[I]n the context of false or misleading health information, we speak of bad (or dangerous) health literacy --, meaning the presence of the ability to understand medical information turned sour by the simultaneous absence of the ability to recognize it as false." (Allam et al [131]).	[131]
